# Single‐cell functional analysis of parathyroid adenomas reveals distinct classes of calcium sensing behaviour in primary hyperparathyroidism

**DOI:** 10.1111/jcmm.12732

**Published:** 2015-12-05

**Authors:** James Koh, Joyce A. Hogue, Yuli Wang, Matthew DiSalvo, Nancy L. Allbritton, Yuhong Shi, John A. Olson, Julie A. Sosa

**Affiliations:** ^1^ Department of SurgeryDuke University Medical CenterDurhamNCUSA; ^2^ Department of Biomedical EngineeringUniversity of North CarolinaChapel HillNCUSA; ^3^ North Carolina State UniversityRaleighNCUSA; ^4^ Departments of Surgery and BiochemistryUniversity of Maryland School of MedicineBaltimoreMDUSA; ^5^ Departments of Surgery and Molecular BiologyUniversity of Maryland School of MedicineBaltimoreMDUSA; ^6^ Duke Cancer Institute and Duke Clinical Research InstituteDuke University Medical CenterDurhamNCUSA

**Keywords:** hyperparathyroidism, parathyroid, adenoma, calcium, single‐cell analysis

## Abstract

Primary hyperparathyroidism (PHPT) is a common endocrine neoplastic disorder caused by a failure of calcium sensing secondary to tumour development in one or more of the parathyroid glands. Parathyroid adenomas are comprised of distinct cellular subpopulations of variable clonal status that exhibit differing degrees of calcium responsiveness. To gain a clearer understanding of the relationship among cellular identity, tumour composition and clinical biochemistry in PHPT, we developed a novel single cell platform for quantitative evaluation of calcium sensing behaviour in freshly resected human parathyroid tumour cells. Live‐cell intracellular calcium flux was visualized through Fluo‐4‐AM epifluorescence, followed by *in situ* immunofluorescence detection of the calcium sensing receptor (CASR), a central component in the extracellular calcium signalling pathway. The reactivity of individual parathyroid tumour cells to extracellular calcium stimulus was highly variable, with discrete kinetic response patterns observed both between and among parathyroid tumour samples. CASR abundance was not an obligate determinant of calcium responsiveness. Calcium EC50 values from a series of parathyroid adenomas revealed that the tumours segregated into two distinct categories. One group manifested a mean EC50 of 2.40 mM (95% CI: 2.37–2.41), closely aligned to the established normal range. The second group was less responsive to calcium stimulus, with a mean EC50 of 3.61 mM (95% CI: 3.45–3.95). This binary distribution indicates the existence of a previously unappreciated biochemical sub‐classification of PHPT tumours, possibly reflecting distinct etiological mechanisms. Recognition of quantitative differences in calcium sensing could have important implications for the clinical management of PHPT.

## Introduction

Primary hyperparathyroidism (PHPT) is a common endocrine neoplastic disorder, with an overall incidence of 15–30 per 100,000 individuals in the USA. 1, 2, 3. The disease disproportionately affects the elderly, with a prevalence rate of 99 affected individuals per 100,000 among women aged 65–74 years, a substantial clinical burden that is projected to expand significantly as the nation's median age rises 4, 5. The morbidity of PHPT can be significant, including bone loss and fracture, nephrolithiasis, cardiovascular and gastrointestinal disease, and neurocognitive impairment 6. These symptoms arise secondary to a metabolic disturbance in calcium homeostasis imparted by dysregulated parathyroid hormone (PTH) secretion due to a failure of calcium sensing in culprit adenomatous or hyperplastic parathyroid glands 7. Silencing or inactivation of the calcium sensing receptor (CASR) coincident with the emergence of parathyroid neoplasia is the presumptive primary mechanism for the loss of calcium sensing in PHPT 8, 9, 10, 11. However, CASR genetic lesions are not found in sporadic PHPT 12, 13, 14, and multiple lines of evidence from our laboratory 15 and others 16, 17, 18, 19, 20 indicate that tumour aggregate CASR abundance is not predictive of relative calcium responsiveness. Moreover, we have recently shown that parathyroid adenomas are comprised of functionally distinct cellular subtypes that differ in their relative sensitivity to calcium stimulation despite equivalent levels of CASR expression in each population 21. This evidence of intratumoural heterogeneity in the composition and biochemical behaviour of parathyroid adenomas calls into question the assumption of CASR down‐regulation as an obligate mechanism in PHPT and highlights the need for a means to interrogate intrinsic calcium responsiveness of individual parathyroid tumour cells as a more direct functional readout of the underlying calcium sensing deficit.

To investigate how intratumoural heterogeneity affects the calcium sensing behaviour of parathyroid tumours, we sought to examine the dynamic response of parathyroid adenoma cells to extracellular calcium challenge at single cell resolution. Here, we describe a novel, optically transparent, indexed, micron scale live‐cell observation system that allows us to visualize and quantitate the calcium responsiveness of individual cells isolated from surgically resected human parathyroid adenomas. Using this device, a dynamic profile of each cell's intracellular signalling response following extracellular calcium stimulus can be captured as a functional readout of calcium sensing behaviour. The cells can subsequently be probed by immunofluorescence *in situ* or recovered individually for downstream molecular analysis. Utilizing the system's capacity to align post‐assay immunofluorescence image analysis with the individual record of each cell's functional response, we show that the presence of detectable CASR protein is not the sole determinant of calcium sensitivity in parathyroid adenoma cells. We report that *ex vivo* provocative testing to evaluate the biochemical responsiveness of a series of parathyroid adenoma cell isolates across a range of calcium doses reveals five distinct kinetic patterns of calcium flux response. Distribution among these categories at lower calcium concentrations is highly variable between tumours but consolidates into either a maximal response or non‐response profile as calcium concentrations increased. When we plotted the dose**–**response relationship between the proportion of maximal responders and calcium concentration, we found that the adenomas in our sample group appeared to segregate into two distinct categories with respect to calcium EC50. The emergence of these biochemically defined subclasses of parathyroid tumours suggests a previously unappreciated degree of functional heterogeneity in PHPT.

## Materials and methods

### Microfabrication

Microraft arrays were fabricated as previously reported 22. Briefly, UV photolithography was used to fabricate arrays of 50 × 50 × 40 μm 1002F photoresist pillars on a glass substrate. Soft lithography was used to mold polydimethylsiloxane (PDMS) microwell arrays from the templates. The PDMS microwell arrays were then dip‐coated in a solution of poly(styrene‐co‐acrylic acid) doped with Fe_2_O_3_ nanoparticles in γ‐butyrolactone, leaving a bead of the poly(styrene‐co‐acrylic acid) solution inside each PDMS microwell. After baking off the solvent, concave microrafts remained within the microwells. The completed arrays were attached to four‐chambered computerized numerical control (CNC)‐milled polycarbonate cassettes by plasma‐treating both surfaces and then adhering them together using PDMS. In order to enhance surface wettability, the microarrays attached to milled polycarbonate cassettes were plasma‐treated for 5 min., sterilized with ethanol, and coated with glucose. Completed array dimensions are as follows: 26 × 26 mm exterior; 50 × 50 μm^2^ rafts; 10 μm gaps between rafts. Each microraft array contains approximately 180,000 rafts.

### Cell culture

Parathyroid cell suspensions were prepared from surgically resected adenoma tissue as previously described 21. Briefly, the procedure was as follows: the tissue specimens were rinsed in serum‐free DMEM (cat. no. 11995073; Life Technologies, Grand Island, NY, USA) supplemented with an antibiotic/antimycotic solution (cat. no. A5955; Sigma‐Aldrich, St. Louis, MO, USA). In a laminar flow biosafety cabinet (Biosafety Class II; Baker Company, Sanford, ME, USA), the tissue was minced into 1–2 mm pieces using sterile instruments and then rinsed with serum‐free DMEM. The tissue fragments were transferred to a sterile flask containing DMEM with 1 mg/ml collagenase (Type 1A, cat. no. C2671; Sigma‐Aldrich) and allowed to digest with constant stirring in a humidified tissue culture incubator (Forma Model 3110; Thermo Scientific, Marietta, OH, USA) set to 37°C and 5% CO_2_. Stirring occurred on a sealed magnetic stirrer (cat. no. 0003671000; IKA Works, Wilmington, NC, USA) placed inside the tissue culture incubator with the electrical cord passing to an external outlet through a rear‐facing port sealed with a gas‐tight silicone gasket. Collagenase digestion was allowed to proceed until >80% of the tissue was reduced to a mixture of single cells and dispersed cell clumps (approximately 3–4 hrs on average). The cell suspension was pelleted and resuspended in Liu primary cell media 23 lacking the rho kinase inhibitor but containing 5% foetal bovine serum to neutralize the collagenase. The primary cell media described by Liu and co‐workers incorporates the Rho kinase inhibitor Y‐27632 (cat. no. ALX‐270‐333; Enzo Life Sciences, Farmingdale, NY, USA) at a concentration of 5–10 micromolar to support long‐term sustained culture of primary epithelial cells. We have found that the Y‐27632 agent is not required for parathyroid adenoma cell culture in our system and thus we do not include it our media. The cells were filtered through a 50 μm mesh to remove fibrous tissue and were then plated at a density of 5 × 10^4^ cells/cm^2^. Calcium flux assays were initiated within 48 hrs of the time of harvest, well before parathyroid cell viability in culture begins to decline (typically at 7–14 days).

### Calcium flux assay

Parathyroid cell cultures were harvested using Detachin (cat. no. T100110; Genlantis, San Diego, CA, USA), and an aliquot was viewed on a haemacytometer to estimate cell number and verify viability. The cells were washed with low calcium (0.22 mM) Liu media, seeded into the microraft grids and incubated overnight. The calcium flux imaging assay was performed using the Fluo‐4 NW calcium assay reagent (cat. no. F36206; Life Technologies). The reagent was prepared according to the manufacturer's instructions. Cells in the microraft wells were washed once with calcium‐free buffer and were then incubated for 60 min. in Fluo‐4‐containing media to load the cells with the indicator.

Microraft grids were placed in a live‐cell epifluorescence imaging system consisting of a Zeiss Axio Observer (Carl Zeiss AG, Oberkochen, Germany) with a Hamamatsu Orca ER high resolution CCD camera and a heated Z1 motorized stage within a humidified/5% CO_2_ enclosure. After a brief baseline stabilization period, matched sets of identically seeded grid chambers from each adenoma were challenged with a calcium chloride stimulus across a range of final calcium concentrations from 0.5 to 10 mM. Metamorph (v7.8) software (Molecular Devices, Inc, Sunnyvale, CA, USA) in multichannel acquisition mode was used to capture sequential fluorescence images every 5 sec. for 10 min., yielding a time‐lapse image stack of 121 sequential frames for each of three separate fields per calcium concentration point. Flux measurements were collected from a minimum of 200 cells at each calcium concentration point. A brightfield phase contrast image of each field was captured at the beginning and at the end of the 10‐min. observation period for alignment. The exposure time for each fluorescence frame was set to 100 msec., with the brightfield channel exposure set to 50 msec.

### Immunofluorescence

Immediately after completion of the flux assay measurements, the media was removed, and the cells were washed twice with PBS. The cells were then fixed in 3.7% paraformaldehyde (PFA) for 10 min., followed by three washes in PBS. The fixed cells were permeabilized with 0.5% Triton X‐100 in PBS for 15 min. and then washed again with PBS. After blocking in 3% bovine serum albumin (BSA)/PBS for 1 hr at room temperature, the cells were incubated with a mouse monoclonal anti‐CASR primary antibody (Clone 5C10, cat. no. NB120‐19347; Novus, Littleton, CO, USA) at a 1:1000 dilution. Isotype‐matched mouse IgG at the same concentration was used as a negative control. After washing, CASR reactivity was visualized using an AlexaFluor488‐conjugated goat anti‐mouse secondary antibody diluted 1:1000 in 3% BSA/PBS. The secondary antibody incubation was followed by three washes, and then the stained cells were overlaid with Prolong Gold antiquenching mounting media containing DAPI as a nuclear counterstain. Background subtracted anti‐CASR immunfluorescence was quantitated as integrated intensity (fluorescence intensity in the AlexaFluor488 emission wavelength summed over all of the pixels within each cell boundary) for each cell using the Metamorph region measurement tool.

### Image analysis

Background subtraction was performed using the multichannel review mode of Metamorph. Individual cells were marked manually as numbered regions using a standardized template. Fluorescence above background was calculated as integrated intensity for each cell region in each of the 121 sequential images in each image stack using the Metamorph region measurement tool. Raw data including region area, average intensity, peak intensity, integrated intensity above background, well location and related parameters were exported directly in Excel spreadsheets. Fluorescence intensities were normalized to each cell's zero time‐point value and kinetic response curves were plotted over the full time course of each experiment. Kinetic profile categories are defined as follows. The maximal response profile requires a sharp increase in induced calcium flux within the first 60 sec. of Ca^2+^ exposure followed by a sustained (<10% intensity deviation) plateau of fluorescence intensity for the duration of the remaining observation period. The rapid/transient profile shows an immediate increase in fluorescence intensity within 60 sec. of calcium stimulus with a return to baseline within the subsequent 60‐sec. time interval. Slow responders show a prolonged interval of fluorescent intensity increase of >2 min. before achieving a plateau phase. Multiple peak responders have at least two fluorescence maxima separated by a return to baseline in the interpeak interval. Non‐responders show a <10% deviation from baseline over the entire observation period.

### Procurement of patient material

Parathyroid cells were isolated from surgically resected adenoma tissue obtained from patients with PHPT undergoing parathyroidectomy at Duke University Medical Center. Tissue identity as parathyroid adenoma was established by intraoperative PTH monitoring using the Miami criteria 24 of a >50% decline from pre‐operative PTH levels within 10 min. of tumour resection, and confirmed by post‐operative histopathological assessment of the surgical specimen. All procedures on human subjects were reviewed and approved by the Duke University Institutional Review Board (IRB). Patients pre‐operatively diagnosed with PHPT were recruited by endocrine surgery and enrolled in the study after providing fully informed consent as described under an active IRB‐approved protocol. De‐identified parathyroid adenoma surgical specimens were provided to the laboratory for immediate harvest and recovery of live dispersed parathyroid cells as described above.

## Results

**Figure 1:**
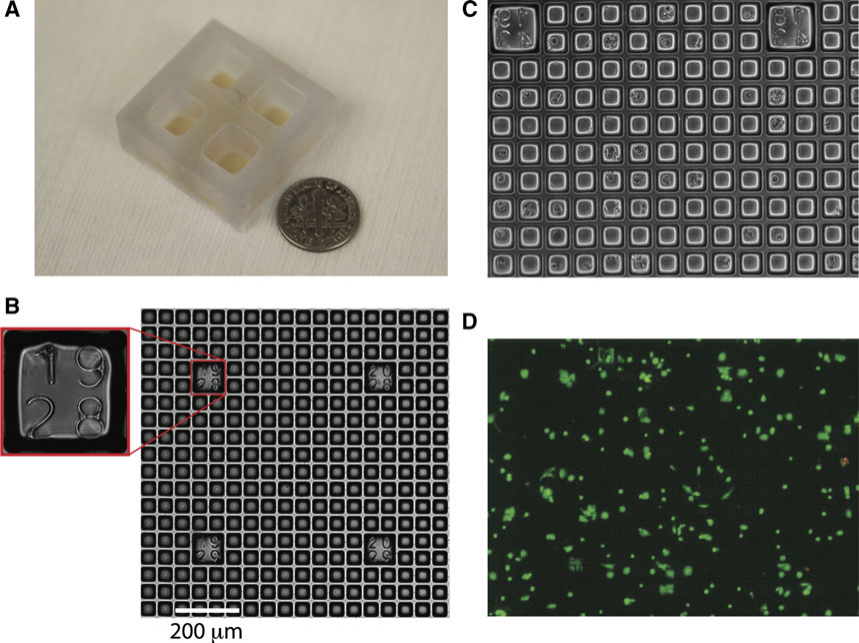
Flexible micro‐raft design. (**A**) Photograph of the microraft array platform module with four independent grid chambers. (**B**) Transmitted light microscopy image of a microraft array with numeric indices identifying the spatial location of the field of view within the array; scale bar: 200 μm. (**C**) Phase contrast image of parathyroid cells adherently plated in a microraft grid array. (**D**) Parathyroid cell viability after plating. Viable cells are indicated by Oregon green staining; non‐viable cells are marked by propidium iodide staining (red).

The novel live‐cell imaging approach we developed for quantitatively interrogating the dynamic calcium responsiveness of human parathyroid tumour specimens at single cell resolution couples detection of an intracellular calcium flux indicator (Fluo‐4‐AM) with a unique plating array device. In this system, parathyroid adenoma cells are seeded in an optically transparent indexed grid array composed of a large number of releasable μm‐scale elements, termed microrafts, doped with paramagnetic nanoparticles 25, 26, 27. Four independent grid chambers are held in a CNC‐milled polycarbonate block that can be mounted in a standard inverted microscope mechanized stage (Fig. 1A). Each 50 × 50 μm^2^ microraft constitutes a discrete and separable culture site with a unique address within the indexed grid array (Fig. 1B). Dispersed parathyroid cell suspensions prepared from surgically resected human parathyroid adenomas using previously established methods 21 adhered well to the microraft surface (Fig. 1C) and maintained viability at >96% in culture (Fig. 1D).

**Figure 2:**
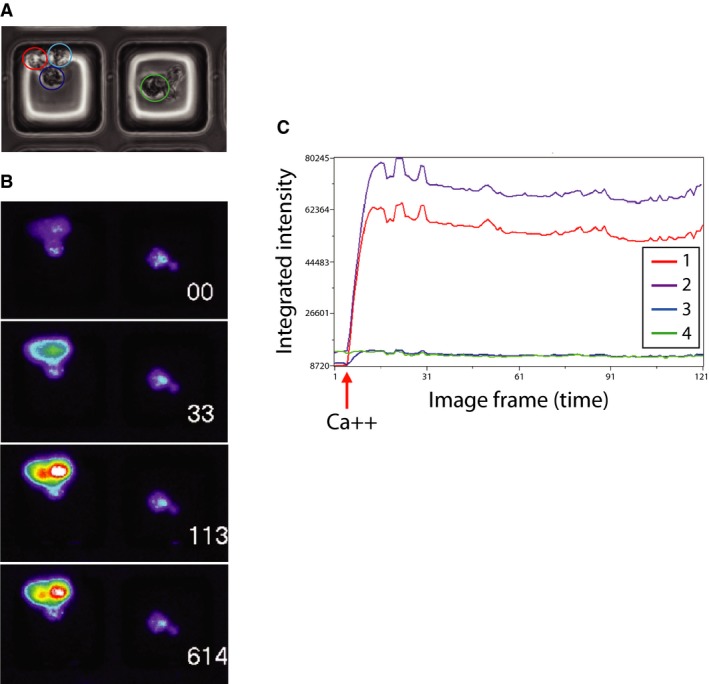
Intracellular flux response of parathyroid cells. (**A**) Phase contrast image of parathyroid cells in two adjacent micro‐raft wells. (**B**) Sequential fluorescent images of the same field of cells in (**A**) after 2 mM calcium stimulus. White numerals indicate number of seconds after stimulus. Increasing fluorescence intensity is indicated using a standard pseudo‐colour heat map scale. Numbered circles denote individual cell regions. (**C**) Quantitative record of calcium flux in the four individually marked cells. Flux curve line colours and numbering match the region boundary colours and numbering in (**B**).

The viability of the dispersed cell isolates was additionally documented by propidium iodide exclusion at the end of the calcium flux assay to confirm the sustained integrity of the primary cells through the duration of the experimental observation period (Table S1). Expression of PTH, CASR, MEN1 and STAMBP was visualized by immunofluorescence to confirm parathyroid identity (Fig. S1). Parathyroid adenoma cells plated into microraft grid arrays were interrogated functionally for responsiveness to extracellular calcium challenge. An example of comparative single cell calcium flux response is shown in Figure 2, where a cluster of four cells in two adjacent microrafts are found to display markedly different levels of calcium response. The cells appear morphologically indistinguishable under brightfield illumination (Fig. 2A), but upon stimulation with 2 mM CaCl_2_, two of the cells respond strongly while the remaining two cells are non‐responsive (Fig. 2B). A plot of integrated fluorescence intensity over time for these cells reveals the quantitatively distinct flux patterns manifested by the responsive (1 and 2) relative to the non‐responsive (3 and 4) cells in this image field (Fig. 2C). To verify that non‐responsiveness in this assay was not due to inadequate uptake of the fluo‐4‐AM indicator or artifactual depletion of intracellular calcium stores, we stimulated the cells with the calcium ionophore ionomycin at the conclusion of the primary response observation period. Ionomycin‐induced intracellular flux was uniformly detected regardless of the initial calcium response level of the cells (Fig. S2), verifying the specificity of the calcium stimulation outcome.

**Figure 3:**
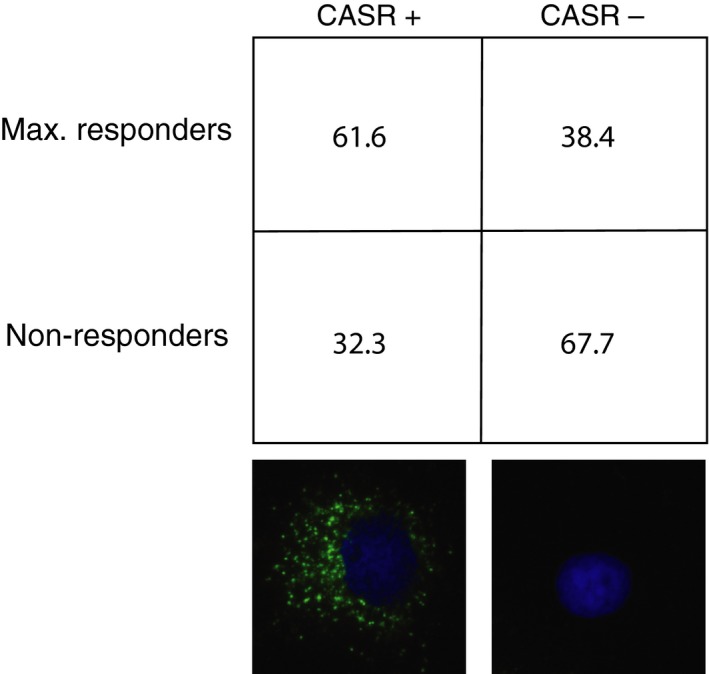
Relationship of calcium responsiveness and CASR expression. Distribution of cells in each category is expressed as mean percentage ± S.D. from four independent patient samples. Immunofluorescence images of representative CASR+ and CASR− cells below each respective column. Green = anti‐CASR; blue = DAPI.

We initially suspected that expression of the CASR could be the determinant factor that distinguished calcium‐responsive from non‐responsive cells. To test this idea, we performed calcium flux measurements in a series of primary parathyroid adenoma specimens and then probed the cells for CASR protein expression *via* immunofluorescence *in situ* on the microraft grids. Anti‐CASR immunofluorescence intensity was not predictive of calcium responsiveness. The mean ± S.D. CASR intensity value (pixel intensity per cell) for responsive cells was 2.809 ± 0.474 [*n* = 285; 95% confidence interval (CI): 2.221–3.397], while the intensity value for non‐responsive cells was 2.574 ± 1.220 (*n* = 180; 95% CI: 1.968–3.181). The *P*‐value for difference between the mean CASR intensity values in responsive and non‐responsive cells was 0.625. The relatively wide CIs for CASR expression among responsive and non‐responsive cells suggested that CASR‐positive cells could be found in both groups. Aggregate data from four different adenoma specimens support this notion. While CASR‐expressing cells were enriched among responders (790 CASR‐positive cells/1283 responsive cells), 38.4% of calcium‐responsive cells did not express detectable CASR protein (493 CASR‐negative cells/1283 responsive cells), and conversely, 32.3% of non‐responding cells did express CASR (883 CASR‐positive cells/2732 non‐responsive cells) (Table S2). The mean ± S.D. proportion of cells in each category (CASR‐positive/responsive, CASR‐positive/non‐responsive, CASR‐negative/responsive, CASR‐negative/non‐responsive) for the four specimens demonstrates that CASR‐negative/responsive and CASR‐positive/non‐responsive cells are consistently observed among the four samples tested (Fig. 3). These data suggest that CASR expression is not the sole determinant of calcium responsiveness in parathyroid adenoma cells.

**Figure 4:**
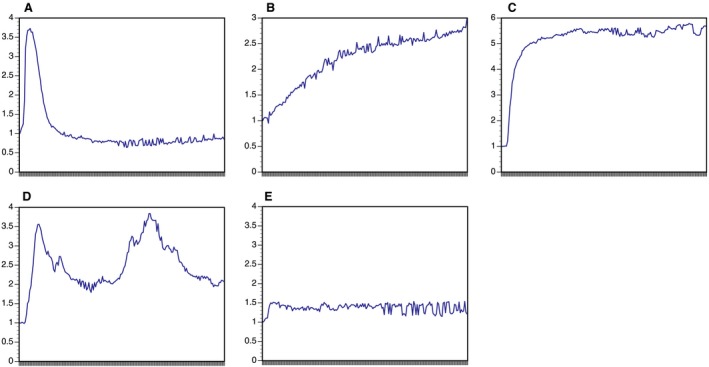
Kinetic response patterns of parathyroid cells after calcium stimulus. Mean fluorescence intensity (MFI) is shown on the *y*‐axis; observation time (10 min. total) is shown on the *x*‐axis. (**A**) Rapid/transient response. (**B**) Slowly rising response. (**C**) Maximal response. (**D**) Multiple peak response. (**E**) Non‐responsive.

**Figure 5:**
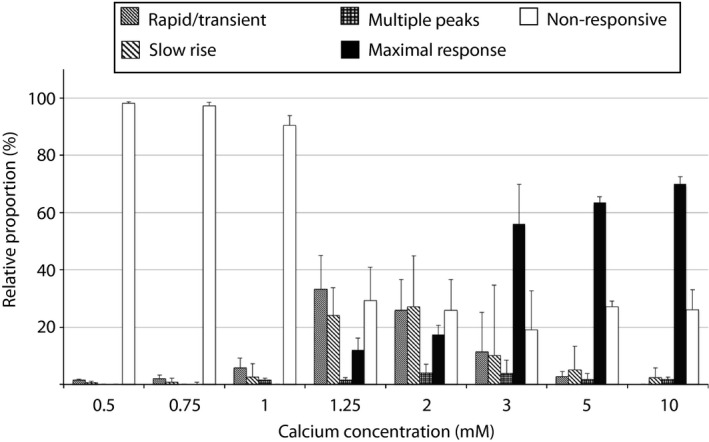
Proportional distribution of cells in five kinetic response categories at different calcium concentrations. Bars indicate mean proportion ± S.D. from 11 adenoma samples.

We observed that the kinetic profiles of calcium responsiveness among parathyroid tumour cells could be classified into five temporally distinct patterns (Fig. 4). Responsive cells displayed either a rapid, transient flux peak (Fig. 4A), a slowly rising flux increase (Fig. 4B), a sustained maximal response (Fig. 4C) or multiple flux peaks (Fig. 4D). Non‐responsive cells showed minimal flux after calcium stimulus and represent the fifth profile pattern (Fig. 4E). Distribution of adenoma cells among these categories was heterogeneous (Table S3) and highly variable at or just above physiological calcium concentration levels (1.25 and 2 mM) but progressively consolidated into either the non‐responsive or maximally responsive categories at higher calcium concentrations (>3 mM) (Fig. 5, Table S4). Non‐responsive cells predominated at sub‐physiological (<1.25 mM) calcium levels.

**Figure 6:**
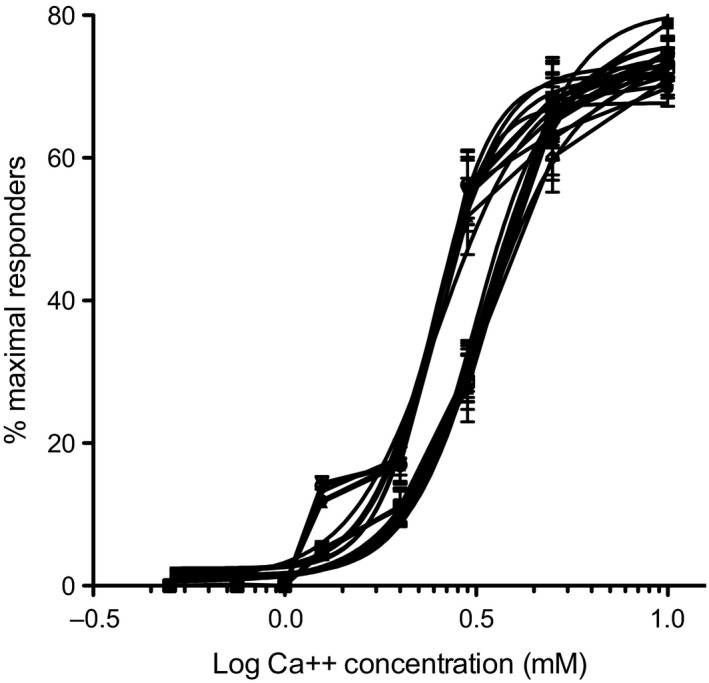
Dose**–**response curves depicting proportion of maximally responsive cells as a function of log calcium concentration. Data points represent mean ± S.D. from three independent fields of >200 cells/field for each calcium concentration from each patient sample. Traces of 11 adenoma samples are shown.

Because the proportion of cells manifesting a maximal response profile appeared to increase in a concentration dependent manner (Fig. 5, Fig. S3 and Table S4), we reasoned that quantitative analysis of this functional biochemical relationship could reveal compromised calcium sensitivity in parathyroid adenomas. To examine the dose**–**response relationship between the proportion of maximally responsive cells in a given tumour and extracellular calcium concentration, we performed a series of flux measurements in 11 consecutive primary parathyroid adenoma specimens. The cells were challenged with a zero calcium media control or with extracellular calcium to a final concentration of 0.5, 0.75, 1, 1.25, 2, 3, 5 or 10 mM, and the proportion of maximally responsive cells was recorded. The normalized data were plotted as a function of log calcium concentration and fitted into a standard dose**–**response curve using a variable slope four‐parameter logistic model. As shown in Figure 6, the adenomas tested appeared to segregate into two distinct groups with respect to calcium sensitivity. The mean calcium EC50 of the first group was 2.40 mM (95% CI: 2.37–2.41 mM), which is closely aligned with the published sensitivity conferred by wild‐type CASR 20. The calcium EC50 for the second group was 3.61 mM (95% CI: 3.45–3.95 mM), which is indicative of attenuated calcium responsiveness. The difference between the mean calcium EC50 values was highly significant, with a *P* < 0.0001.

## Discussion

Intratumoural heterogeneity is widely recognized as a critical factor in nearly all human solid tumours 28, 29, 30, but the lack of streamlined tools for examining live‐cell functional diversity in real time from human tumour tissue remains a significant translational challenge. Current methodologies based on static classification of fixed human tissue or on murine modelling using genetically engineered induced tumours or patient‐derived xenograft approaches are clearly informative but limited by a number of important considerations. Genomic profiling coupled with histopathological analysis is a powerful approach for molecular classification, but it does not directly measure dynamic function. Murine systems relying on candidate gene manipulation may not reflect the multifactorial origin, heterogeneous cellular composition, and complex physiological behaviour of native tumours. Patient‐derived xenograft models provide a better context for evaluating tumour heterogeneity, but experiments using these systems are expensive, labour‐intensive, and time‐consuming. Here, we describe a simple, *ex vivo* approach for dynamic functional interrogation of live parathyroid adenoma cells collected directly from surgically resected human tumour tissue. By evaluating the functional behaviour of these cells at single‐cell resolution, we can quantitate the effects of intratumoural heterogeneity on calcium responsiveness, the key physiological activity underlying the clinical presentation of PHPT. Because our platform utilizes direct observation of individual cellular behaviours, the system can easily be adapted for examining specimens where cell numbers are limiting. *Ex vivo* provocative testing of human tumour tissue in this manner could potentially provide improved diagnostic precision and functional insight into the dynamic biochemical behaviour of multiple types of human solid tumours.

Our approach does carry certain limitations that could affect interpretation of these experiments. It is possible that selective cellular recovery or survival could create biased representation in the cellular populations seeded into the grids. Although we cannot rule out the potential for biased recovery, collagenase digestion is an established procedure for cellular harvest from intact tissue; in addition, we have not observed consistent digestion‐resistant structures, and the ultrastructural morphology of cells isolated in this manner matches their appearance in intact tissue 21. We feel that selective cell death is unlikely to be a significant factor, as the overall viability of parathyroid cells in our system is consistently >90% (Fig. 1D). We recognize that positional information and tumour microenvironment context are not preserved in dispersed cell systems and that appropriate subcellular trafficking and activity of the CASR protein might be at least partially dependent upon intact tissue structure. We have in fact observed intratumoural heterogeneity in CASR protein localization (Fig. S4), although the overall proportion of CASR‐positive and CASR‐negative cells in the microraft arrays is consistent with what we observe in tissue sections from the parental tumours. We are currently developing a modified device to allow visualization of calcium flux behaviour in intact parathyroid adenoma slice cultures.

Our observation that CASR protein abundance alone does not dictate calcium responsiveness supports the concept of mechanistic heterogeneity in the failure of calcium sensing in parathyroid adenomas. Alterations in a number of proteins that operate in the CASR signal transduction pathway have been associated with disruptions of calcium sensing. For example, in previous work we have shown that increased expression of the G‐protein regulatory protein RGS5 can attenuate calcium sensing in human cells and in a transgenic mouse model 15. Conversely, germ‐line inactivating mutations in the guanine nucleotide binding protein G‐alpha11 or in the protein‐trafficking complex protein AP2S1 cause the calcium sensing disorders of familial hypocalciuric hypercalcemia Types II and III, respectively 19, 20. Somatic mutations in these genes have not been reported in PHPT 12, 13, 14, but this could be because the relevant subset of functionally non‐responsive cells was underrepresented in the bulk tumour samples used in the genomic studies published to date. Future studies utilizing selective microraft capture of functionally characterized parathyroid adenoma cells are likely to reveal molecular genetic features specifically associated with diminished calcium sensing even in the presence of intact CASR protein expression.

The variable kinetic patterns of adenoma cell responsiveness to extracellular calcium challenge reveal yet another level of intratumoural heterogeneity in these complex tumours. The greatest degree of diversity in the proportional distribution among kinetic response profiles occurs at or near physiological calcium concentrations, suggesting that the presence of the full spectrum of signalling patterns represents an equilibrium state in parathyroid gland tissue. Consolidation into a binary distribution of maximally responsive or non‐responsive cells at higher calcium concentrations suggests that the calcium signalling response of parathyroid cells is saturable in a manner similar to that observed in cultured cells stably expressing wild‐type CASR protein 31, where higher calcium concentrations induce a sustained elevation of intracellular calcium release 32. Heterogeneity in the calcium response profiles of parathyroid cells from a given sample could indicate the co‐existence of subpopulations with different calcium setpoints within each tumour. The respective functions and interactions of these differing cellular subsets remain unknown but are the subject of active investigation.

Although the initial sample group in the current study (Table S5) is too small to support any conclusive inferences on possible associations between biochemical behaviour and clinical parameters, the observed variation in tumour cell calcium responsiveness evokes a number of intriguing hypotheses for further investigation. The apparent segregation of an unselected series of PHPT adenomas into separable groups on the basis of calcium EC50 values suggests the existence of biochemically distinct subclasses of PHPT, possibly reflecting differing etiological mechanisms. The fact that one group displays calcium responsiveness similar to the reported value for wild‐type CASR implies that a subset of parathyroid tumours may be intrinsically capable of appropriate calcium sensing to some degree. It is possible that these normal‐sensing tumours arise in a manner more akin to secondary HPT, where hyperplasia is driven by metabolic conditions or other events that do not directly compromise calcium sensing. In PHPT adenomas with normal calcium sensitivity, the presence of an initiating genetic lesion could have driven clonal expansion of a parathyroid tumour without necessarily impinging upon intrinsic calcium responsiveness. In contrast, polyclonal expansion of a parathyroid tumour could occur as a consequence of failed calcium sensing, with tumour growth directly linked to abrogated CASR activity as has been demonstrated in other tumour types 33, 34. The extent of association between clonal status and calcium EC50 is currently being tested (Fig. S5). Ongoing efforts to establish definitive linkage between parathyroid adenoma cell functional properties and clinical parameters, disease course and outcome will require larger patient cohorts drawn from a systematically assembled parathyroid tumour registry database.

In summary, functional characterization of parathyroid intratumoural heterogeneity with respect to calcium sensing reveals a previously unappreciated degree of complexity in the etiopathogenesis and clinical diversity of PHPT. The development of a live‐cell observation platform that can align dynamic interrogation of functional behaviour with subsequent immunofluorescence image analysis and future downstream genomic profiling at single cell resolution provides a valuable new tool for dissecting the molecular basis of compromised calcium sensing in PHPT. The concept of live‐cell functional interrogation as a means of tumour classification could have important implications for the development of improved prognostic and predictive clinical management guidelines.

## Conflicts of interest

N.L.A. discloses a financial interest in Cell Microsystems, a licensee of the microraft technology used in this manuscript. The other authors have no competing interests.

## Supporting information


**Figure S1** Immunofluorescence detection of marker expression in dispersed parathyroid cells.

**Figure S2** Ionomycin‐stimulated intracellular calcium release.

**Figure S3** Proportion of maximal responder cells at different calcium concentrations.

**Figure S4** Intratumoural heterogeneity in CASR protein subcellular localization.

**Figure S5** Dose**–**response curves from two parathyroid adenomas with known clonal status.

**Table S1** Parathyroid adenoma dispersed cell viability.
**Table S2** Association of CASR expression and calcium responsiveness in four adenoma samples.
**Table S3** Distribution of flux response kinetic profiles in samples from three parathyroid adenomas.

**Table S4** Distribution of cells in five kinetic response categories at increasing calcium concentrations in a representative parathyroid adenoma sample.

**Table S5** Patient characteristics.
