# Supplementary figures and images for: Single‐cell functional analysis of parathyroid adenomas reveals distinct classes of calcium sensing behaviour in primary hyperparathyroidism
Source: J Cell Mol Med. 2015 Dec 5;20(2):351–9. doi: 10.1111/jcmm.12732 (PMC4727552; doi:10.1111/jcmm.12732)

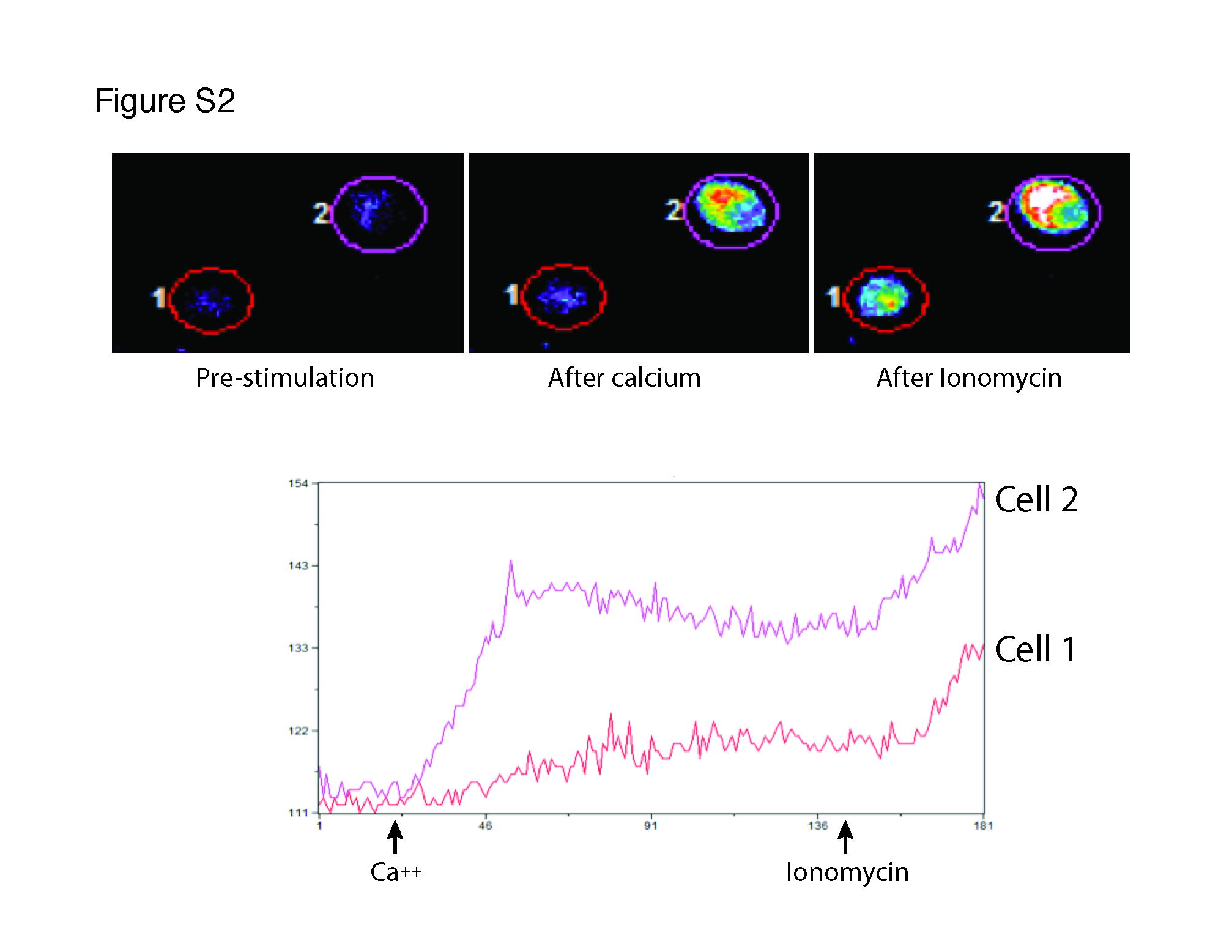

Supplement: Supplementary file 2 — Figure S2 Ionomycin‐stimulated intracellular calcium release. [file JCMM-20-351-s002.tiff]

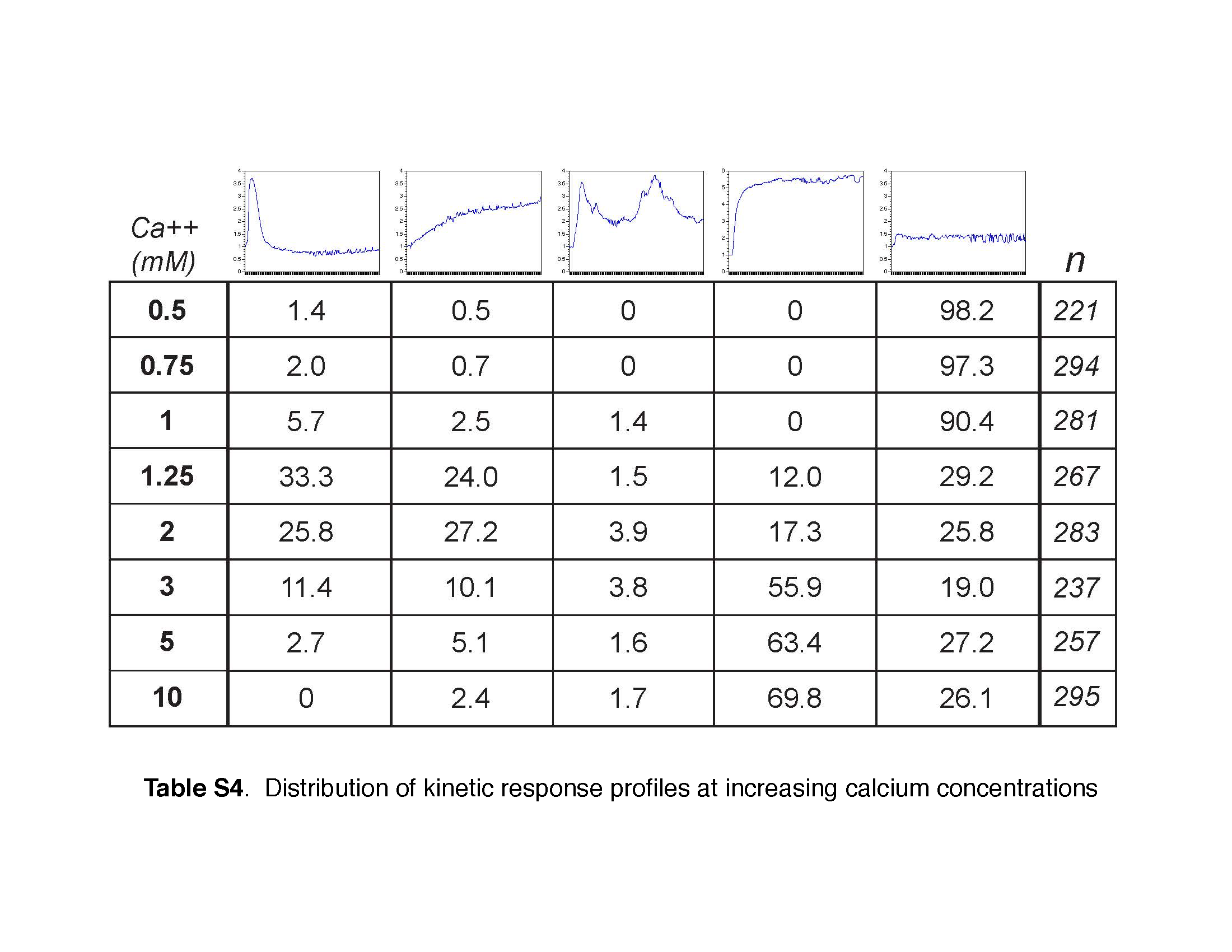

Supplement: Supplementary file 7 — Table S4 Distribution of cells in five kinetic response categories at increasing calcium concentrations in a representative parathyroid adenoma sample. [file JCMM-20-351-s007.tiff]
