# Supplementary material for: Single‐cell functional analysis of parathyroid adenomas reveals distinct classes of calcium sensing behaviour in primary hyperparathyroidism
Source: J Cell Mol Med. 2015 Dec 5;20(2):351–9. doi: 10.1111/jcmm.12732 (PMC4727552; doi:10.1111/jcmm.12732)
Supplement: Supplementary file 6 — Table S1 Parathyroid adenoma dispersed cell viability. Table S2 Association of CASR expression and calcium responsiveness in four adenoma samples. Table S3 Distribution of flux response kinetic profiles in samples from three parathyroid adenomas. [file JCMM-20-351-s006.docx]

|  | **PT 095** | | **PT 013** | | **PT 089** | | **PT 104** | | **PT 094** | | **PT 090** | |
| --- | --- | --- | --- | --- | --- | --- | --- | --- | --- | --- | --- | --- |
|  | Dead | Live | Dead | Live | Dead | Live | Dead | Live | Dead | Live | Dead | Live |
| Field 1 | 12 | 114 | 6 | 105 | 11 | 151 | 6 | 88 | 9 | 112 | 10 | 88 |
| Field 2 | 10 | 108 | 5 | 97 | 8 | 133 | 3 | 112 | 6 | 158 | 4 | 94 |
| Field 3 | 11 | 124 | 7 | 172 | 8 | 153 | 7 | 102 | 7 | 119 | 11 | 96 |
|  |  |  |  |  |  |  |  |  |  |  |  |  |
| Total | 21 | 346 | 18 | 374 | 27 | 437 | 16 | 302 | 22 | 389 | 25 | 278 |
| % Viable | 94.3 | | 95.4 | | 94.2 | | 95.0 | | 94.6 | | 91.7 | |

Table S1. Parathyroid adenoma dispersed cell viability.

| **Patient 1** | **CASR +** | **CASR -­‐** |
| --- | --- | --- |
| Responsive (n=253) | 64.4 | 35.6 |
| Non-­‐responsive (n=260) | 54.2 | 45.8 |
|  |  |  |
| **Patient 2** |  |  |
| Responsive (n=359) | 70.2 | 29.8 |
| Non-­‐responsive (n=1004) | 30.0 | 70.0 |
|  |  |  |
| **Patient 3** |  |  |
| Responsive (n=172) | 45.9 | 54.1 |
| Non-­‐responsive (n=279) | 15.8 | 84.2 |
|  |  |  |
| **Patient 4** |  |  |
| Responsive (n=499) | 59.3 | 40.7 |
| Non-­‐responsive (n=1189) | 33.4 | 66.6 |

Table S2. CASR expression and calcium responsiveness


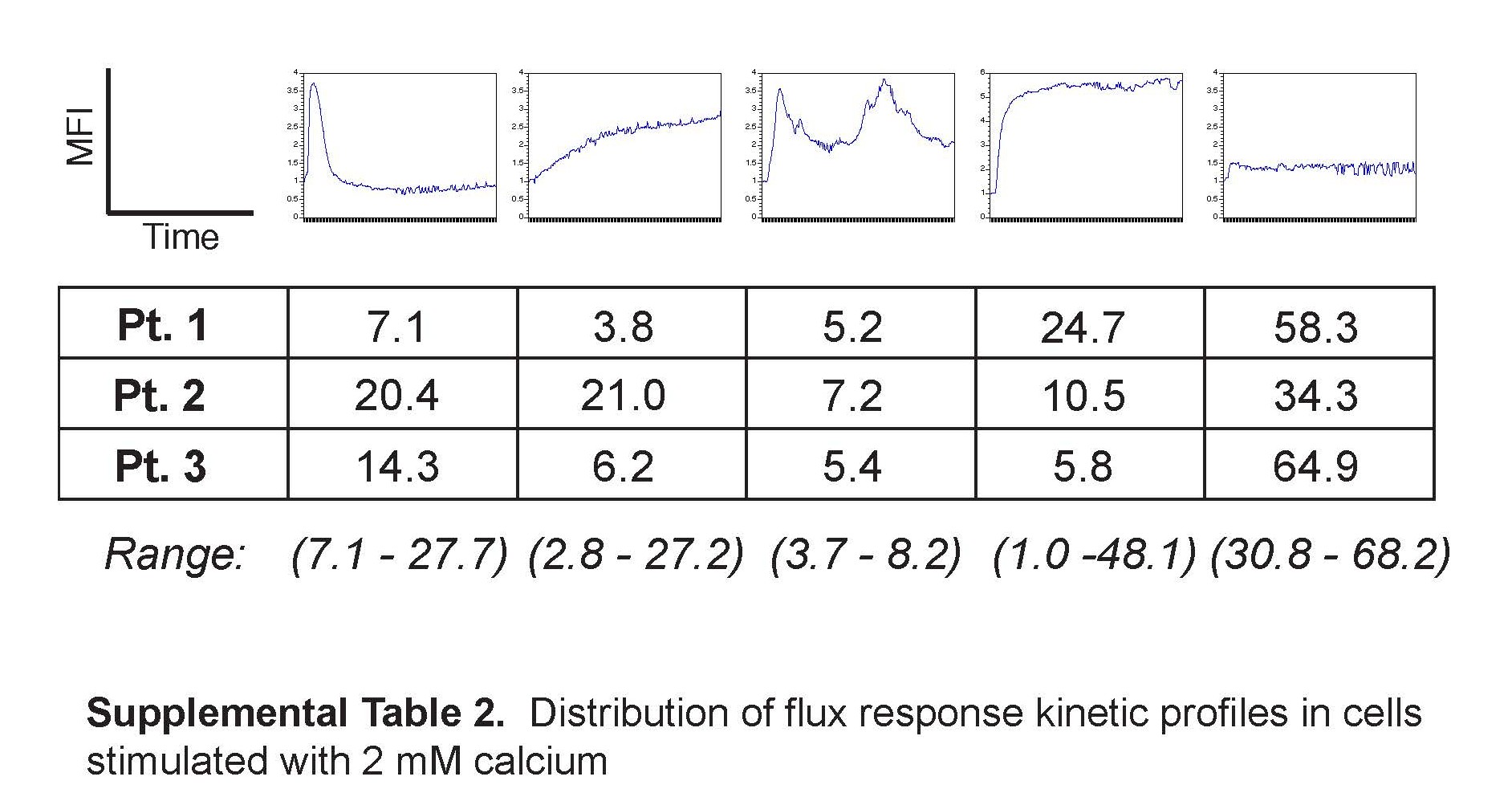


Table S3. Distribution of flux response kinetic profiles in cells stimulated with 2 mM calcium

' .: .:0 ' " "VV ""V '

·'':

Ca++

'

.:.:

:( "

' ' ' '

'' HI 1 " 1J'IIiVWf\ "

' '

*(mM)* '·' ' ' " "  *n*

| **0.5** | 1.4 | 0.5 | 0 | 0 | 98.2 | *221* |
| --- | --- | --- | --- | --- | --- | --- |
| **0.75** | 2.0 | 0.7 | 0 | 0 | 97.3 | *294* |
| **1** | 5.7 | 2.5 | 1.4 | 0 | 90.4 | *281* |
| **1.25** | 33.3 | 24.0 | 1.5 | 12.0 | 29.2 | *267* |
| 2 | 25.8 | 27.2 | 3.9 | 17.3 | 25.8 | *283* |
| 3 | 11.4 | 10.1 | 3.8 | 55.9 | 19.0 | *237* |
| **5** | 2.7 | 5.1 | 1.6 | 63.4 | 27.2 | 257 |
| **10** | 0 | 2.4 | 1.7 | 69.8 | 26.1 | *295* |

' '

' ' ' ' '

**Table 54.** Distribution of kinetic response profiles at increasing calcium concentrations
