# Supplementary material for: Single‐cell functional analysis of parathyroid adenomas reveals distinct classes of calcium sensing behaviour in primary hyperparathyroidism
Source: J Cell Mol Med. 2015 Dec 5;20(2):351–9. doi: 10.1111/jcmm.12732 (PMC4727552; doi:10.1111/jcmm.12732)
Supplement: Supplementary file 8 — Table S5 Patient characteristics. [file JCMM-20-351-s008.docx]

| **Patient Number** | **Age** | **Gender** | **Pathology** | **Pre-operative calcium (mg/dL)**  *Normal range: 8.5 -10.2* | **Pre-operative PTH (pg/ml)**  *Normal range: 10 - 65* | **Calcium EC50 (mM)** |
| --- | --- | --- | --- | --- | --- | --- |
| 30 | 71 | F | Single adenoma | 11.5 | 69 | 2.45 |
| 32 | 47 | F | Single adenoma | 11 | 132 | 2.42 |
| 35 | 65 | F | Single adenoma | 11.7 | 140 | 3.25 |
| 38 | 45 | F | Single adenoma | 11.7 | 183 | 2.45 |
| 43 | 62 | M | Double adenoma | 13.6 | 540 | 3.25 |
| 44 | 61 | F | Single adenoma | 9.8 | 283 | 3.23 |
| 45 | 65 | M | Single adenoma | 10.9 | 67 | 2.46 |
| 46 | 50 | M | Single adenoma | 11.4 | 100 | 2.39 |
| 48 | 79 | F | Single adenoma | 10.4 | 76 | 3.18 |
| 52 | 73 | F | Single adenoma | 10.3 | 116 | 3.42 |
| 58 | 75 | F | Single adenoma | 11.3 | 260 | 3.14 |

**Table S5**. Patient characteristics. Patient numbers are anonymous identifier codes issued for each study participant. None of the patients had a family history of parathyroid disease. Pre-operative clinical biochemistry values are obtained from the parathyroid tumor clinical registry database maintained by our research group. Calcium EC50 values were generated as described in the text.
