# Supplementary material for: Single‐cell functional analysis of parathyroid adenomas reveals distinct classes of calcium sensing behaviour in primary hyperparathyroidism
Source: J Cell Mol Med. 2015 Dec 5;20(2):351–9. doi: 10.1111/jcmm.12732 (PMC4727552; doi:10.1111/jcmm.12732)
Supplement: Supplementary file 9 [file JCMM-20-351-s009.docx]

**Table S1.** *Parathyroid adenoma dispersed cell viability*. Cell counts from Live (Hoechst 33342 positive) vs Dead (propidium iodide positive) cells were scored from 3 different microscope fields from six different adenoma specimens at the end of the calcium flux response assay.

**Figure S1.** *Immunofluorescence detection of marker expression in dispersed parathyroid cells.* Primary human parathyroid cells were fixed, permeabilized, and stained with primary antibodies against MEN1, STAMBP, PTH, CASR, or RGS5 (upper row) or with negative control IgG matched to the species origin of the primary antibody (lower row). Ms IgG = mouse Ig G; RbIgG = rabbit IgG; GtIgG = goat IgG. Immunoreactivity was visualized through fluorochrome-conjugated secondary antibodies. Secondary antibody labels were anti-rabbit AF488 for MEN1 and STAMBP, anti-mouse AF488 for PTH and CASR, and anti-chicken AF568 for RGS5. Scale bar = 20 microns. Images were captured with a 20X (MEN1, CASR) or 40X (RGS5, PTH) objective lens.

**Figure S2.** *Ionomycin-stimulated intracellular calcium release*. At the conclusion of the calcium-response assay observation period, the cells were exposed to 1 uM ionomycin to provoke intracellular calcium release. Fluorescence channel images captured before calcium stimulation (at frame 1), after calcium stimulation (frame 46), or after ionomycin stimulation (frame 181) are shown. The image frames are from a total sequence of 181 frames taken at 5-second intervals. The graph shows a plot of fluorescence intensity (y-axis) over time (x-axis) for the two cells shown in the image frame series. Arrows indicate the time of addition for calcium stimulation or ionomycin treatment.

**Table S2.** *Association of CASR expression and calcium responsiveness in four adenoma samples.* The proportions of CASR-positive and CASR-negative cells among responsive or non-responsive parathyroid cells from four patients are shown.

**Table S3.** *Distribution of flux response kinetic profiles in samples from three parathyroid adenomas*. Numbers in the table represent percentages of total evaluated cells stimulated with 2 mM calcium.

**Table S4.** *Distribution of cells in five kinetic response categories at increasing calcium concentrations in a representative parathyroid adenoma sample.* Characteristic traces for each of the five response categories are shown above columns listing the proportion of cells in each category at different calcium concentrations (rows). Calcium stimulus in mM is shown in bold in the far left column. Total numbers of cells counted at each calcium concentration are shown in the far right column.

**Figure S3.** *Proportion of maximal responder cells at different calcium concentrations.* Bars depict the mean +/- standard deviation for the percentage of cells from 11 adenomas displaying the maximal response profile at different calcium concentrations.

**Figure S4**. *Intratumoral heterogeneity in CASR protein subcellular localization.* CASR immunofluorescence staining images from two different regions of the same parathyroid adenoma are shown. CASR reactivity is shown in green. Nuclear staining is shown in blue. Inset: CASR localization in normal parathyroid tissue.

**Figure S5.** *Dose-response curves from two parathyroid adenomas with known clonal status.* The calcium EC50 for the clonal adenoma was 2.39 mM (95% CI: 2.37–2.40, n=2135). The polyclonal adenoma EC50 was 3.42 mM (95% CI: 3.40–3.44, n=2042).
